# Specialized pro-resolving mediators in myocardial infarction: orchestrators of inflammation resolution and tissue repair

**DOI:** 10.3389/fcvm.2026.1728794

**Published:** 2026-03-24

**Authors:** Yating Jiang, Liwen Du

**Affiliations:** Emergency Department, Ningbo No.2 Hospital, Wenzhou Medical University, Ningbo, China

**Keywords:** cardioprotection, inflammation, inflammation-resolution signalling, myocardial infraction, pro-resolving mediators

## Abstract

Myocardial infarction (MI) remains a leading cause of morbidity and mortality wordwide. Despite reperfusion therapies have improved survival, persistent and dysregulated inflammation contributes to adverse remodeling and progression to heart failure. However, the mechanisms by which inflammation is actively terminated and converted into a reparative program in the infarcted heart have not been fully elucidated. Specialized pro-resolving mediators (SPMs)—including lipoxins, resolvins, protectins and maresins—represent a unique class of lipid-derived mediators that regulate the active resolution of inflammation without broadly suppressing host immune defenses like conventional anti-inflammatory drugs. Emerging evidence demonstrates that SPMs can regulate neutrophil clearance, promote efferocytosis, modulate endothelial activation and reprogram cardiac macrophages toward a reparative phenotype. Notably, SPMs signaling interracts with multiple cores signaling networks involved in MI pathology, including the NF-κB, AMPK, Hippo-YAP and JAK/STAT pathways, linking metabolic states and inflammatory signals to structural repair processes. Despite these advances, critical gaps remain regarding the temporal dynamics of SPM biosynthesis after MI, their mechanistic interactions with existing standard therapies, and their clinical translation as biomarkers or therapeutic agents. This review integrates the latest mechanistic and clinical evidence to propose a spatiotemporal specific SPMs-driven cardiac repair framework and highlights how targeting endogenous resolution pathways may complement current MI management. By dissecting key molecular nodes within SPMs signaling, we propose a therapeutic strategy that extend beyond “inflammation suppression” to actively restoring myocardial homeostasis.

## 1 Introduction

Myocardial infarction(MI) is an acute cardiovascular event characterized by irreversible myocardial injury resulting from abrupt reduction or cessation of coronary blood flow (1). Its hallmark pathological features include cardiomyocyte necrosis and progressive deterioration of left ventricular structure and function (2, 3). Despite advances in reperfusion therapies such as percutaneous coronary intervention (PCI), the global incidence and the mortality rates of MI continue to rise (4).

Following MI, the inflammatory response progresses through temporally coordinated yet partially overlapping phases: an initial stage dominated by neutrophil infiltration and cytokine release, followed by gradually transitions into a phase in which inflammation resolution and tissue repair proceed in parallel (5, 6). While the inflammatory response is essential for initiating repair, conventional anti-inflammatory strategies that broadly suppress immunity may impede the clearance of dead cells and tissue debris, ultimately leading to poor outcomes (7). Therefore, precise regulation of the inflammatory response is critical for optimal cardiac repair after MI (8).

In recent years, a unique class of endogenous bioactive lipids and proteins, known as specialized pro-resolving mediators (SPMs), have gained increasing attention (9–12). Unlike conventional anti-inflammatory agents, SPMs actively resolve inflammation by modulating immune cell phenotypes (13, 14). These mediators do not simply suppress inflammation but actively reprogram the inflammatory microenvironment and accelerate its resolution. These mediators enhance efferocytosis (15, 16), limit neutrophil infiltration and reprogram macrophages toward an anti-inflammatory pro-resolving phenotype, promoting tissue homeostasis without impairing host defense (17, 18). SPMs are particularly promising for MI (10), where timely resolution of inflammation and tissue regeneration are essential for preventing chronic complications, such as heart failure (8, 19).

However, several challenges hinder the clinical translation of SPMs-based therapies (20), including an incomplete understanding of their spatiotemporal signaling networks, difficulties in achieving targeted delivery, and a lack of biomarkers to monitor resolution responses (8, 21). This review discusses the molecular mechanisms by which SPMs regulate inflammation resolution and tissue remodeling during both the acute and chronic phases of MI, and emerging therapeutic strategies to harness SPMs for improving clinical outcomes.

During the acute phase (1–5 days), MI triggers necrotic cardiomyocyte death and the release of damage-associated molecular patterns (DAMPs), which activate pattern-recognition receptors and initiate NF-κB–dependent inflammatory cascades. Neutrophils rapidly infiltrate the infarcted myocardium in response to chemokines (CXCL1/2) and adhesion molecules (ICAM1, VCAM1), release reactive oxygen species (ROS), neutrophil extracellular traps (NETs), and matrix-remodeling enzymes that exacerbating cardiomyocyte death, which in turn triggers the release of DAMPs (HMGB1), inflammatory cytokines (IL-1β, TNF-α), and matrix degradation. Ly6C^high^ monocytes are recruited and differentiate into proinflammatory M1 macrophages, amplifying tissue injury and sustaining cytokine production. As inflammation progresses, SPMs, including lipoxins, resolvins and maresins etc. are biosynthesized through lipoxygenase pathways and primarily exert their actions by engaging G protein–coupled receptors on macrophages (such as ALX/FPR2, GPR32, ChemR23, and LGR6). SPMs suppress neutrophil recruitment, enhance efferocytosis, inhibit excessive fibroblast activation and aberrant extracellular matrix remodeling. And they can directly attenuate cardiomyocyte apoptosis. Collectively, these actions promote effective inflammation resolution and restoration of myocardial homeostasis. The lower panel depicts the temporal evolution of pro-inflammatory, anti-inflammatory, and pro-resolving pathways, highlighting their dynamic overlap throughout the post-MI response.

## 2 The inflammatory response to myocardial infarction

**Figure 1:**
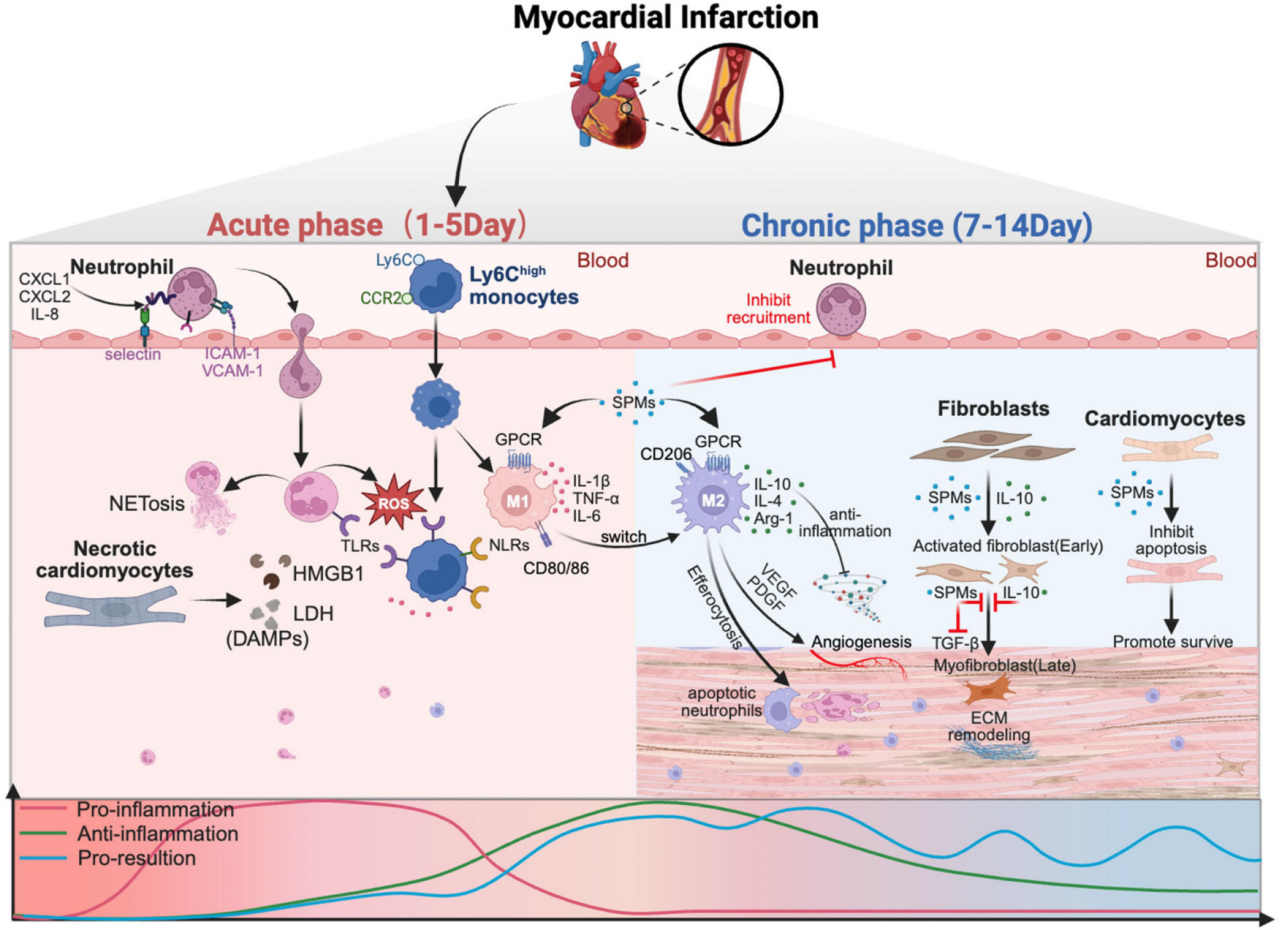
Temporal dynamics of inflammation, immune cell responses, and tissue remodeling after myocardial infarction.Created in BioRender. Ya ting, J. (2026) https://BioRender.com/xc34u3t, licensed under Academic License.

MI triggers a complex inflammatory response that progresses through two phases: the acute inflammatory phase (days 1–5) and the chronic reparative phase (days 7–14) (6, 22, 23). These phases are crucial for tissue healing, with immune cell regulation and signaling pathways driving the transition from injury to repair (Figure 1).

### 2.1 Inflammatory initiation and innate immune activation

Upon myocardial injury, necrotic cardiomyocytes release DAMPs like HMGB1 and S100A8/9, which activate pattern recognition receptors (PRRs) on immune cells, including monocytes and macrophages (24–26). This initiates an immune response characterized by neutrophils and inflammatory monocytes infiltration into the infarcted region. Neutrophils, recruited by chemokines, release proteolytic enzymes, reactive oxygen species (ROS) and neutrophil extracellular traps (NETs) (27, 28), exacerbating myocardial injury and inflammation (29–31). Ly6C^high^ monocytes extravasate into the infarcted myocardium and differentiate into pro-inflammatory M1 macrophages, which secrete pro-inflammatory cytokines (32, 33). Persistent M1 polarization contributes to oxidative damage and tissue breakdown (34–37).

### 2.2 Initiation of active resolution programs and tissue repair

As inflammation subsides, the myocardium enters the reparative phase (6). The biosynthesis of SPMs is enhanced through the activation and transformation of immune cells: (1) macrophages gradually transition to an M2 phenotype in the inflammatory microenvironment, producing SPMs (38); dendritic cells, through the interaction with cytokines and other stimuli, increase the synthesis of SPMs, helping to maintain tissue homeostasis (8); T cells, especially Th2 and Treg cells, also produce SPMs that regulate immune responses and tissue repair (39). (2) immune cells directly release anti-inflammatory cytokines such as IL-4, IL-10 and TGF-β, which not only inhibit the release of inflammatory mediators but also regulate cellular metabolism, promoting the production of SPMs. Subsequently, SPMs signal through specific receptors to coordinate a series of pro-resolving events. Key steps include: **(1) Limiting neutrophil recruitment:** SPMs such as Lipoxin A4 (LXA4) can reduce further neutrophil infiltration into the infarct area. **(2) Promoting efferocytosis:** SPMs enhance the phagocytic clearance of apoptotic neutrophils and necrotic debris by macrophages, which is a critical step in inflammation resolution. This process itself can generate anti-inflammatory and pro-repair mediators, such as resolvins, protectins, IL-10 and TGF-β. **(3) Macrophage phenotypic reprogramming:** Following MI, macrophages undergo an orchestrated phenotypic change during cardiac repair: in the early phase, macrophages derived from recruited inflammatory monocytes, displaying robust phagocytic activity and secretion of pro-inflammatory mediators; subsequently, under the influence of signals from the injured microenvironment, macrophages actively respond to SPMs, gradually shifting phenotypes toward promoting efferocytosis, angiogenesis and tissue remodeling. These repair-functional macrophages secrete anti-inflammatory cytokines, pro-angiogenic factors and mediators involved in ECM remodeling, while continuing to clear cellular debris (23, 40, 41). **(4) Facilitating tissue repair and remodeling:** Under the influence of SPMs, fibroblasts differentiate into myofibroblasts, depositing ECM proteins to stabilize the infarct region (38, 42, 43). Appropriate angiogenesis supports the healing process. This phase is crucial for preventing pathological fibrosis and preserving cardiac function (44, 45). Therefore, post-MI healing largely depends on the delicate balance and timely coordination between pro-inflammatory signals and active pro-resolving signals, with SPMs acting as the central commanders of this resolution program (46).

## 3 Types and mechanisms of SPMs

**Figure 2:**
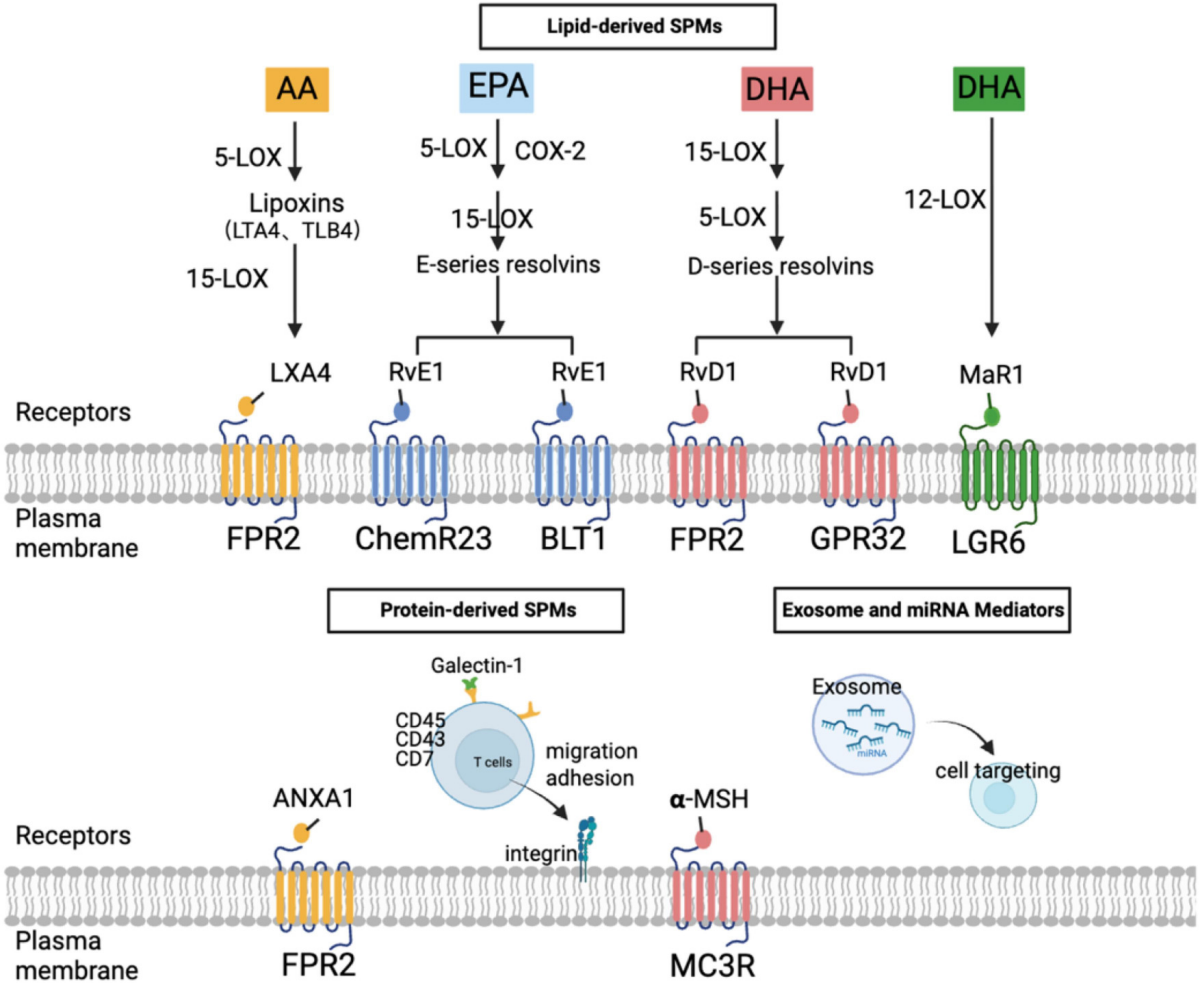
Biosynthesis pathways and receptors of SPMs. Lipid-derived SPMs are synthesized from PUFAs via lipoxygenases (LOXs) and cyclooxygenases (COXs) pathways. Lipoxins, such as LXA4, are derived from arachidonic acid (AA); Resolvins such as RvE1, are generated from eicosapentaenoic acid (EPA); RvD1 and Maresins (MaR1) are derived from docosahexaenoic acid (DHA). These lipid mediators exert their biological effects mainly through GPCRs, including ALX/FPR2, ChemR23, leukotriene B4 receptor 1(BLT1), GPR32 and LGR6. Protein-derived SPMs include ANXA1 and Galectin-1. In addition, extracellular vesicles such as exosomes and specific microRNAs function as emerging classes of SPMs. Together, these distinct mediator families coordinate the resolution of inflammation and the promotion of tissue repair following MI. Created in BioRender. Ya ting, J. (2026) https://BioRender.com/ynyggkv, licensed under Academic License.

SPMs encompass lipid-derived SPMs, protein-derived SPMs, extracellular vesicles and others. Lipid-derived mediators include resolvins, maresins, lipoxins and others, which act through G-protein-coupled receptors (GPCRs), such as ALX/FPR2, ChemR23 and GPR32. Derived from *ω*-3 polyunsaturated fatty acids, play crucial roles in the resolution of inflammation and tissue repair after MI (Figure 2) (8, 47, 48).

### 3.1 Lipid-derived SPMs

Lipid-derived SPMs are enzymatically generated from ω-3 polyunsaturated fatty acids, primarily eicosapentaenoic acid (EPA) and docosahexaenoic acid (DHA), via the coordinated actions of cyclooxygenases and lipoxygenases within the infracted myocardium (43, 48–51). Their major subclasses—Lipoxins, Resolvins and Maresins—exert distinct yet converging roles in MI by regulating neutrophil infiltration, macrophage efferocytosis and phenotypic switching, and fibroblast activation (8, 43, 52). They act via specific GPCRs, such as ALX/FPR2**,** GPR32 and ChemR23 to initiate pro-resolving programs (9, 43, 53) (Table 1).

**Table 1:** Summary of clinical-related studies on SPMs or their analogs in various diseases and conditions.

Disease or procedure	Mediator	Role	Refs
ST-elevation myocardial infarction	SPM	In STEMI patients, plasma SPM levels are significantly elevated immediately after onset and prior to the peak of troponin T, compared to healthy controls and patients with stable coronary artery disease; these mediator levels gradually decline during follow-up. The acute-phase increase in SPMs is primarily attributed to the elevation of protectins derived from eicosapentaenoic acid (EPA) and docosahexaenoic acid (DHA)	47
Acute myocardial infarction	LXA4	In patients with acute myocardial infarction, higher levels of LXA4 are associated with a lower risk of MACE.	100
Coronary artery disease	SPM	In patients with stable coronary artery disease, levels of several SPMs are reduced. n-3 fatty acids can influence the SPM profile in patients with coronary artery disease and promote thrombus remodeling.	106
Healthy subjects	LXA4	Aspirin initiates the biosynthesis of novel anti-inflammatory mediators (15-epi-lipoxin A4) through the interaction between endothelial cells and leukocytes.	107
Periodontitis	LXA4	In a Phase 1 clinical trial, daily oral irrigation with an LXA4 analog (methyl ester-benzo-LXA4) significantly increased serum LXA4 levels and reduced periodontitis parameters (bleeding and probing depth).	108
Acute pancreatitis	LXA4	Preoperative injection of diclofenac significantly increases serum LXA4 levels and reduces the incidence of acute pancreatitis following endoscopic retrograde pancreatography.	114
—	LXA4 and others	LXA4 and other lipid and non-lipid pro-resolving mediators represent an emerging source of novel therapies for chronic cardiovascular inflammation and atherosclerosis.	101
—	LXA4	Several therapeutic approaches for cardiovascular and metabolic diseases—including aspirin, ticagrelor, statins, and pioglitazone—have been shown to increase circulating levels of LXA4.	116–117

STEMI, ST-elevation myocardial infarction; EPA, eicosapentaenoic acid; DHA, docosahexaenoic acid; LXA4, lipoxin A4; MACE, major adverse cardiovascular event.

**Lipoxin A4 (LXA4):** Synthesized from AA, LTA4 signals through ALX/FPR2 to attenuate neutrophil-mediated damage and promote reparative macrophage polarization in the ischemic heart (43). Preclinical studies demonstrate that LXA4 administration reduces infarct-associated inflammation, limits adverse ventricular remodeling and improves cardiac function (54). Correspondingly, impaired endogenous lipoxin biosynthesis has been observed post-MI, indicating a failure in timely resolution signaling (55). The stable epimer 15-epi-LXA4 has been shown to activate receptors including FPR2 and GPR120. It orchestrates neutrophil recruitment during the acute phase and accelerates their clearance during the resolution phase, thereby attenuating renal inflammation and injury markers such as NGAL and plasma creatinine after MI (55). Moreover, LXA4 and its synthetic analogs promote inflammation resolution and reduce infarct size by activating FPR2 on cardiac macrophages (56).

**Resolvins (RvD1, RvE1):** The RvE series derives from EPA: activated neutrophils convert EPA into 18-HpEPE via 5-LOX. This intermediate is then transformed by acetylated COX-2 or P450 enzymes in neighboring cells into 18R/S-HEPE, leading to the formation of RvE1 and RvE2. Alternatively, within macrophages, 5-LOX and 12/15-LOX can cooperate to directly produce RvE3 from EPA (57–59). The RvD series originates from DHA: neutrophils first convert DHA into 17S-HpDHA via 5-LOX (60). This product is subsequently further oxidized by 12/15-LOX in macrophages or epithelial cells. The site of oxygenation determines the product type: oxygenation at the C7 position yields the 7S, 8S-epoxide intermediate, which hydrolyzes to form RvD1–RvD4; oxygenation at the C4 position generates the 4S, 5S-epoxide intermediate, hydrolyzing into RvD5 and RvD6 (61, 62). Primary RvDs can also be enzymatically modified to produce metabolites with varying biological activities (59). RvD1 exerts anti-inflammatory and pro-resolving effects via ALX/FPR2 and GPR32 (63, 64). In MI, RvD1 reduces infarct size, limits fibroblast activation, and helps stabilizes the nascent ECM by modulating macrophage programming. Pretreatment with RvD1 has been shown to protect against MI by downregulating HMGB1 and its associated TLR4 and NF-*κ*B signaling (65). A clinical study involving 140 subjects (88 STEMI patients and 52 non-cardiac chest pain controls) reported lower serum RvD1 levels in the STEMI group. Low admission levels of RvD1 were closely associated with adverse prognostic markers in STEMI, including high-sensitivity C-reactive protein, pro-B-type natriuretic peptide and troponin (66).

RvE1 mainly acts through ChemR23, protecting cardiomyocytes through AKT activation and suppression of NF-κB signaling (67). It has demonstrated cardioprotective effects against LPS-induced cardiac injury (67), doxorubicin-induced cardiotoxicity (68, 69) and hypertension-induced vascular remodeling (70). *In vivo* experiments demonstrated that RvE1 dose-dependently reduced myocardial infarct area in rats, while *in vitro* experiments showed that RvE1 increased cell viability and decreased apoptosis in a dose-dependent manner (71).

**Maresin1 (MaR1):** Discovered in 2009, maresins are generated via 12-LOX-mediated conversion of DHA (72–74). MaR1 promotes cardiac repair post-MI by enhancing macrophage efferocytosis, improving mitochondrial fitness in cardiomyocytes, reinforcing endothelial barrier integrity, and directly suppressing endoplasmic reticulum stress via PPARα/FOXO1-dependent signaling (75, 76). In experimental MI, MaR1 accelerates inflammatory resolution and enhances clearance of apoptotic neutrophils while modulating fibroblast activation and collagen synthesis, thereby preventing maladaptive scar formation—an emerging SPMs-dependent therapeutic axis in cardiac repair (77).

### 3.2 Protein mediators

Endogenous protein mediators, including ANXA1, Galectin-1 and Melanocortins (α-MSH), exhibit receptor-dependent pro-resolving activities distinct from lipid mediators (78, 79). ANXA1 and its mimetic peptides activate FPR2, limiting neutrophil–endothelial interactions and enhancing macrophage efferocytosis during ischemic injury (80). ANXA1 deficiency aggravates infarct expansion and delays resolution of inflammation, underscoring its central role in MI-associated tissue repair. Galectin-1 regulates leukocyte motility and T-cell apoptosis (81, 82), whereas α-MSH promotes cytokine repression and immune tolerance through MC1R signaling (83). Together, these protein SPMs synergize with lipid mediators to coordinate immune orchestration and prevent sustained pro-inflammatory signaling in the ischemic myocardium.

### 3.3 Exosome and miRNA mediators

Exosome-derived SPMs function as intercellular carriers linking immune and stromal compartments during myocardial healing (84–86). Macrophages incorporate RvD1 and RvD2, into microvesicles, facilitating spatially restricted delivery of resolution signals across the infarcted myocardium (87). microRNAs (miRNAs) within exosomes play distinct roles in modulating the inflammatory milieu. For instance, the miR-146 family can negatively regulate TLR4 and NF-κB signaling pathways while miR-155-containing exosomes from M1 macrophages inhibit angiogenesis and worsen myocardial damage (86, 88, 89).

## 4 The role of SPMs in the acute and chronic phases of MI

**Figure 3:**
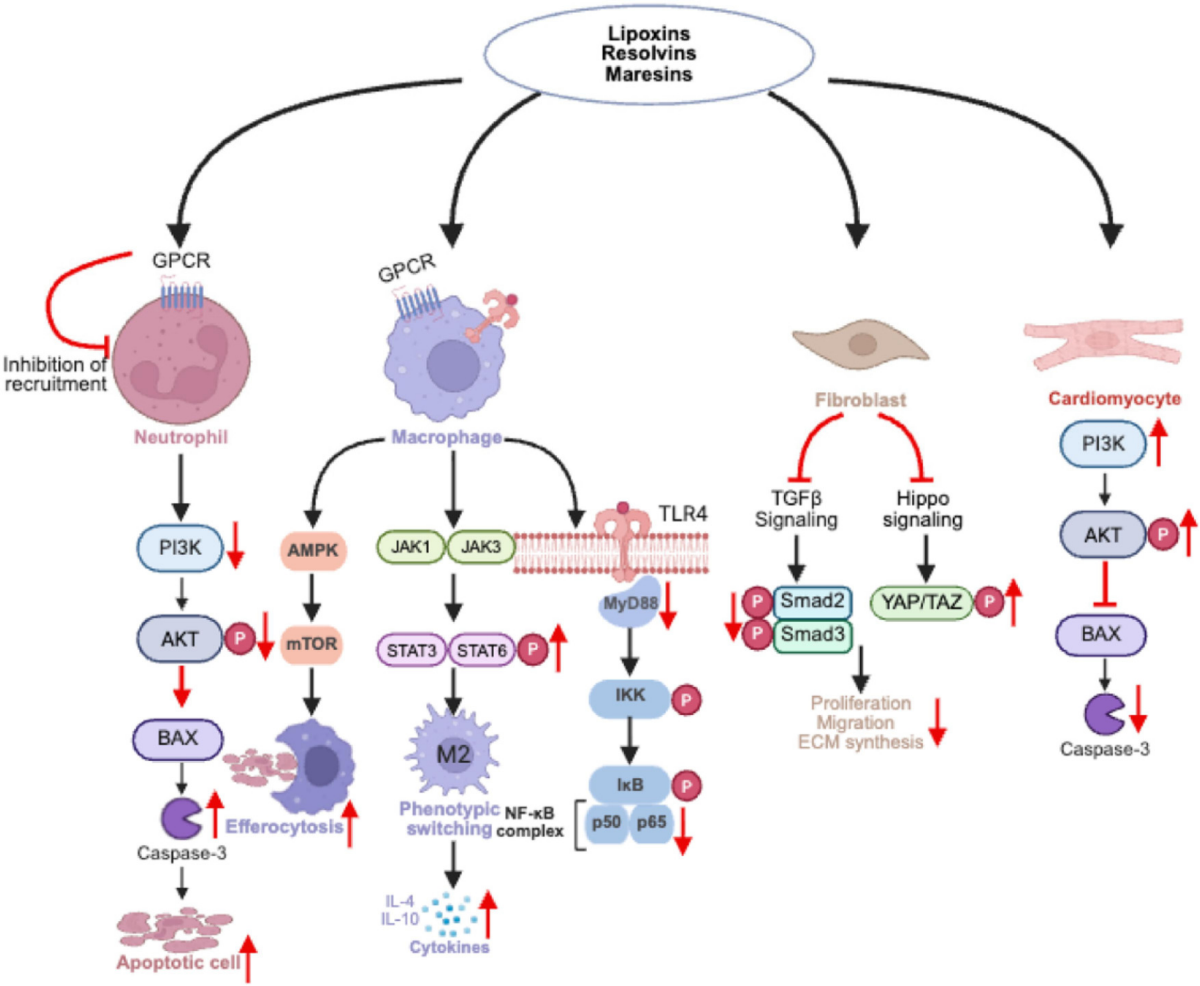
Integrated signaling mechanisms of SPMs during MI. SPMs, such as Lipoxins, Resolvins, and Maresins, regulate various cell functions in MI by acting on specific GPCRs. They inhibit neutrophil recruitment, reduce inflammatory infiltration and suppress BAX and caspase-3 mediated apoptosis via the PI3K/AKT signaling pathway. In macrophages, SPMs promote phenotypic switching, enhance efferocytosis and inhibit NF-κB activation through the JAK/STAT and AMPK/mTOR pathways, thereby reducing pro-inflammatory cytokine secretion and driving a reparative phenotype. In fibroblasts, SPMs modulate the TGF-β/Hippo signaling pathway to limit activation and ECM synthesis, preventing pathological scar formation. In cardiomyocytes, SPMs activate PI3K/AKT signaling to inhibit BAX- and caspase-3-mediated apoptosis, thereby protecting cell survival. Created in BioRender. Ya ting, J. (2026) https://BioRender.com/ejv7tbs, licensed under Academic License.

In the acute phase, SPMs limit neutrophil infiltration, reduce oxidative stress and modulate key signaling pathways like NF-κB and MAPK (Figure 3) (8, 57). For example, RvD1, RvE1 and LXA4 can activate AMPK/mTORC1 signaling. This mechanism not only promotes autophagic flux and the clearance of apoptotic cells but also couples energy sensing with inflammation resolution: AMPK activation enhances the metabolic adaptability of macrophages, promotes mitophagy, and improves their efferocytic capacity, thereby effectively clearing necrotic debris (79). RvD1 engages FPR2 to suppress pro-inflammatory cytokine, enhance macrophage-mediated debris clearance and limit infarct expansion. RvE1 improves cardiac function when administered within the first seven days post-MI by inhibiting Ly6C^high^ monocyte/macrophage recruitment, whereas delayed treatment (7–14 days) has less benefit (32, 90). MaR1 alleviates oxidative stress and inflammation by inhibiting TLR4/MyD88 signal transduction, blocking IKK complex activation and maintaining the stability of IκBα protein. This in turn prevents the nuclear translocation and DNA binding of the transcription factor p50–p65 dimer. The suppression of this signaling cascade leads to a significant reduction in the expression of downstream pro-inflammatory cytokines (such as TNF-α, IL-6, and IL-1β), ultimately mitigating oxidative stress, reducing cardiomyocyte apoptosis, and inhibiting fibrotic progression (72). SPMs also regulate macrophage polarization by activating JAK signaling and promoting STAT6 phosphorylation, thereby driving the functional reprogramming of macrophages from a pro-inflammatory to a reparative state (Figure 3) (91, 92). This shift not only suppresses NF-κB dependent cytokine production but also supports fibroblast regulation and tissue repair. These findings highlight the therapeutic value of early SPMs intervention in MI.

During the chronic phase, SPMs counteract fibrosis by modulating fibroblast activation and ECM remodeling (Figure 3) (8, 93). For example, MaR1 inhibits nuclear translocation of YAP in fibroblasts, reducing collagen synthesis and myofibroblast differentiation (94). RvD1 activate AKT, inhibiting pro-apoptotic proteins such as Caspase-3 and 8, protecting cardiomyocytes and modulating fibroblast function (95). Furthermore, SPMs support neovascularization and myocardial integrity by promoting endothelial cell survival and limiting pathological fibrosis. ALX/FPR2 knockout mice show impaired inflammation resolution and macrophage dysfunction (96), which can be rescued by exogenous LXA4 (97, 98).

Beyond coordinating the resolution of acute and chronic inflammation, SPMs exert a profound influence on suppressing adverse ventricular remodeling and preventing the progression toward heart failure. Studies have shown that exogenous administration of RvD2 significantly improves cardiac functional parameters (LVEF, FS) in post-MI mice, a benefit mechanistically linked to its suppression of excessive inflammation and promotion of organized collagen deposition (99). Mechanistically, MaR1 inhibits the spread of pathological fibrosis and helps maintain ventricular compliance by balancing ECM synthesis and degradation, as well as modulating fibroblast activation states. Meanwhile, LXA4 and its analogs act through the FPR2/ALX receptor to inhibit the expression of pro-fibrotic factors such as TGF-β while protecting cardiomyocytes from sustained inflammation-induced apoptosis, collectively limiting infarct expansion and overall ventricular remodeling (99). Therefore, SPMs serve not only as drivers of local repair but also as pivotal regulators crucial for maintaining the long-term structural and functional integrity of the heart.

The therapeutic efficacy of SPMs arises from their integration of multiple signaling networks (Figure 3). For instance, AMPK links metabolic regulation to inflammation by inhibiting NF-κB, PI3K/Akt promotes cardiomyocyte survival while intersecting with the Hippo pathway to regulate fibroblast activation; and Hippo-YAP signaling acts as a key brake on pathological fibrosis. Through this coordinated modulation, SPMs simultaneously dampen inflammation, promote debris clearance, enhance cell survival, and suppress maladaptive remodeling—ultimately orchestrating a multifaceted reparative response across both acute and chronic stages of MI.

## 5 Clinical research progress and translational prospects

Although most research on SPMs remain preclinical, accumulating clinical evidence has begun to unveil their dynamic changes and potential value during recovery after MI (100).

### 5.1 Clinical evidence as prognostic biomarkers

Clinical observations indicate that MI can activate endogenous SPM biosynthesis. In patients with ST-segment elevation myocardial infarction (STEMI), plasma levels of various SPMs (such as RvD1, RvD2, MaR1 and LXA4) increase significantly during the acute phase (47, 101). Their dynamic trajectories correlate with inflammatory markers like IL-8, suggesting that an early, active resolution response is crucial for suppressing excessive inflammation and improving long-term prognosis (47). The levels of these SPMs carry clear clinical significance: elevated LXA4 is associated with a reduced risk of recurrent ischemia, while higher levels of RvD1 and RvE1 correlate with better cardiac functional recovery and a lower risk of heart failure (100). Furthermore, in patients with stable coronary artery disease, higher SPM levels are linked to an increase in circulating endothelial progenitor cells, implying a potential role in promoting vascular repair (102). These findings collectively establish SPMs as potential prognostic biomarkers for myocardial recovery and long-term outcomes after MI.

### 5.2 Challenges and advances

The pro-repair properties of SPMs make them attractive novel therapeutic targets. Extensive preclinical studies have confirmed that exogenous administration of RvD1, RvE1 or MaR1 can effectively reduce infarct size, improve cardiac function and inhibit adverse remodeling (65, 71, 75). However, translating these findings into clinical therapeutics faces several challenges. (**1**) **Pharmacokinetic hurdles**: Most natural SPMs have short half-lives and are rapidly metabolized and inactivated *in vivo*. This necessitates therapeutic strategies involving continuous infusion, prodrug design, or the development of metabolically stable analogs (43, 103). **(2) Analytical and detection challenges**: SPMs are present at very low concentrations (picomolar levels) in plasma and are chemically unstable, posing significant challenges for accurate quantification. Although liquid chromatography-tandem mass spectrometry (LC-MS/MS) is the gold standard for detection, the lack of uniform standards, standardized protocols, and high costs limit its routine clinical application (104, 105).

Despite positive signals from basic translational research and early clinical exploration, registered clinical trials directly using SPMs or their stable analogs as therapeutic agents in the cardiovascular field are still in their early stages (106–108). The current research focus is shifting from observing SPMs as prognostic biomarkers to exploring their feasibility as therapeutic drugs.

## 6 Discussion

MI remains a leading cause of increasing morbidity and mortality worldwide (109, 110). Despite advances in reperfusion therapy, inflammation plays a pivotal role in myocardial injury and long-term complications (111), and the timely initiation and active resolution of inflammation are critical determinants of repair quality. This review systematically elucidates the central coordinating role of SPMs in this process. Unlike traditional broad-spectrum anti-inflammatory strategies, SPMs do not simply suppress immune responses. Instead, they actively guide the functional reprogramming of immune cells—particularly macrophages—in a spatiotemporally specific manner, transforming the inflammatory microenvironment from a destructive state into a reparative one in an orderly fashion, thereby promoting structural reconstruction while clearing damage (57).

The actions of SPMs span the entire post-MI repair process. In the acute phase, SPMs such as RvD1 and LXA4 directly mitigate secondary myocardial injury by inhibiting excessive neutrophil infiltration and activation and limiting the release of ROS and proteases (99). More importantly, they enhance the efferocytic capacity of macrophages by activating pathways like AMPK and initiate the phenotypic shift of macrophages toward a reparative state, laying the foundation for the repair phase (79). During the chronic phase, mediators like MaR1 and RvD1 suppress the progression of pathological fibrosis by modulating fibroblast activation (inhibiting YAP nuclear translocation) and balancing ECM synthesis and degradation, while simultaneously promoting beneficial angiogenesis, collectively preserving ventricular structural and functional integrity (38, 94).This seamless transition from “damage control” to “structural reconstruction” underscores the central regulatory role of SPMs in the repair process.

This review focuses on the role of SPMs during the acute and reparative phases of MI, but their broader clinical significance may lie in preventing the development and progression of heart failure. As recent studies indicate, pharmacologically enhancing SPM signaling—for instance, by using RvD2 or stable LXA4 analogs—not only improves acute outcomes in animal models but also confers long-term protection of cardiac function and delays the progression of heart failure. This suggests that targeting the resolution pathway may offer a unique intervention opportunity with an “extended therapeutic window”—even after the peak of acute inflammation, timely supplementation with SPMs could still improve long-term prognosis by modulating chronic low-grade inflammation and fibrotic processes.

A crucial yet often overlooked aspect is the interaction between existing standard-of-care drugs and endogenous resolution pathways (112, 113). Studies have shown that non-selective cyclooxygenase (COX) inhibitors, such as certain nonsteroidal anti-inflammatory drugs, may disrupt the metabolism of SPM precursors, thereby inhibiting inflammation resolution (114, 115). This may also explain the cardiovascular risks associated with these agents. In contrast, some current cardiovascular drugs may promote or mimic the beneficial effects of SPMs through distinct mechanisms. For example, beyond their lipid-lowering effects, statins may exert pleiotropic actions by upregulating lipoxygenase expression or generating SPM-like anti-inflammatory effects (116, 117). Meanwhile, SGLT2 inhibitors may confer cardioprotective effects that synergize with resolution pathways by modulating immune cell function and the inflammatory microenvironment (118). Understanding these interactions is critical for optimizing combination therapeutic strategies.

Despite compelling preclinical and clinical evidence, several challenges remain for SPM translation. First, a significant gap remains between animal models and human disease. Immune responses and cardiac pathophysiology differ substantially between species, and most interventional studies use supraphysiological SPMs doses administered prophylactically or immediately post-infarction. Determining optimal treatment timing, dosage, and delivery routes is essential. Second, the pharmacokinetic profiles of native SPMs—including short half-lives and rapid inactivation—pose considerable drug development challenges. Third, the heterogeneity of MI patients, particularly those with comorbidities such as diabetes or advanced age where resolution pathways may be impaired, calls for personalized treatment strategies.

## 7 Conclusion

In summary, specialized pro-resolving mediators (SPMs) represent a unique class of pro-repair mediators. By integrating multiple signaling pathways such as NF-κB and AMPK, they actively guide inflammation resolution and tissue repair in a spatiotemporally specific manner, thereby establishing a repair program that progresses from “damage control” to “structural reconstruction.” However, their clinical translation still faces critical gaps. Future research should focus on: (1) Developing targeted delivery systems (nanoparticles, engineered exosomes or hydrogels) for controlled SPMs release in the infarct area to improve stability and reduce side effects. (2) Investigating the temporal dynamics of SPMs profiles in MI patients to validate their roles as prognostic biomarkers. (3) Exploring the potential of SPMs as adjuvant therapeutic agents to existing treatments. As discussed in Section 5, clinical observations suggest that the cardioprotective effects of ticagrelor and rosuvastatin may partially depend on the production of pro-resolving mediators; further evaluation of the synergistic effects between SPMs and SGLT2 inhibitors, reperfusion strategies, and emerging regenerative therapies is needed. (4) Further elucidating the interactions between SPMs signaling and other inflammatory/fibrotic cascades to identify therapeutic targets.
